# Combining shallow and deep neural networks on pseudo-color enhanced images for digital breast tomosynthesis lesion classification

**DOI:** 10.3389/fdgth.2025.1705044

**Published:** 2026-01-09

**Authors:** Zhikai Yang, Yingqing Liu, Örjan Smedby, Rodrigo Moreno

**Affiliations:** Department of Biomedical Engineering and Health, KTH Royal Institute of Technology, Stockholm, Sweden

**Keywords:** digital breast tomosynthesis, computer aided diagnosis, deep learning, dual-branch network, pseudo-color enhancement

## Abstract

**Introduction:**

The classification of lesion types in Digital Breast Tomosynthesis (DBT) images is crucial for the early diagnosis of breast cancer. However, the task remains challenging due to the complexity of breast tissue and the subtle nature of lesions. To alleviate radiologists’ workload, computer-aided diagnosis (CAD) systems have been developed. The breast lesion regions vary in size and complexity, which leads to performance degradation.

**Methods:**

To tackle this problem, we propose a novel DBT Dual-Net architecture comprising two complementary neural network branches that extract both low-level and high-level features. By fusing different-level feature representations, the model can better capture subtle structure. Furthermore, we introduced a pseudo-color enhancement procedure to improve the visibility of lesions on DBT. Moreover, most existing DBT classification studies rely on two-dimensional (2D) slice-level analysis, neglecting the rich three-dimensional (3D) spatial context within DBT volumes. To address this limitation, we used majority voting for image-level classification from predictions across slices.

**Results:**

We evaluated our method on a public DBT dataset and compared its performance with several existing classification approaches. The results showed that our method outperforms baseline models.

**Discussion:**

The use of pseudo-color enhancement, extracting high and low-level features and inter-slice majority voting proposed method is effective for lesion classification in DBT. The code is available at https://github.com/xiaoerlaigeid/DBT-Dual-Net.

## 1 Introduction

Breast cancer remains one of the life-threatening diseases in women (1). Among the diagnostic imaging modalities, mammography has long been established as the standard screening method. Despite its widespread use, traditional mammography has obvious limitations, particularly in dense breast tissue, where overlapping structures can obscure lesions or lead to false positives (2). Digital breast tomosynthesis (DBT) is an imaging method that reconstructs a 3-dimensional (3D) volume of the entire breast using a series of low-dose 2D X-ray projections captured from various angles. It retains spatial information across the *Z* direction, improving the detection of tumors in dense tissues (3). Furthermore, DBT improves detailed visualization of breast structures, which could reduce the false positive rate. However, by producing 3D image data, it may increase the radiologists’ workload.

With the development of computer-aided diagnosis (CAD) techniques, many radiomics have been applied in medical diagnosis, demonstrating potential for classifying breast tumors in DBT images (4–6). For example, (7) used linear regression analysis to explore the relationship between radiomics features in DBT images and tumors. (8) evaluated multiple machine learning methods for DBT tumor classification. Their approaches also utilized radiomics features to enable comprehensive image analysis. Several studies explored the relationship between radiomics features from DBT images and the Ki-67 expression level to predict tumor characteristics (9–11). These features may include handcrafted features like morphological, gray-level statistics, and texture features, or contain features extracted with deep learning.

The analysis of DBT images using radiomics techniques remain time-consuming, as it require radiologists to manually delineate suspicious regions. Recently, the application of deep learning in CAD for DBT has gained traction. Numerous studies indicate that deep learning holds significant promise for the classification of breast lesions (12–21).

To tackle the complexity of breast lesions, several studies adopted various approaches to pre-process the dataset. Some studies used separate slices as inputs and report performance at the slice level (13, 15, 16, 18, 19, 22). Kassis et al. (16) employed a vision transformer (ViT) to extract features from 2D DBT slices, followed by post-processing that incorporated information from adjacent slices to classify the entire 3D image. Zhang et al. (21) proposed a novel architecture that includes early fusion and late fusion on multiple-slice 2D DBT images. Early fusion refers to averaging multiple slice images to create a new image, while late fusion involves using a feature extractor to extract features from multiple slices, and then applying a pooling layer to obtain a single feature map. Their study made use of all the information from an individual patient’s lesion case. To fully leverage 3D volumetric information while addressing the challenge of variable slice counts across cases, (17) proposed a preprocessing approach that utilizes a fixed-size (a subset of sections) to replace the complete 3D volume by incorporating a ViT-based network model.

However, the whole-image methods lack interpretability because they do not show a clear causal region to support the prediction. Therefore, some studies directly used region of interest (ROI) lesion patches as inputs. Some researchers have adopted pre-trained results from mammogram ROI data for DBT patch classification tasks using transfer learning (23–25). Samala et al. (24) compared the performance of two approaches with fine-tuning: one was with a multi-stage model, where the network was first fine-tuned on mammograms after pretraining on ImageNet and then further fine-tuned on DBT data, whereas the other was with a model pretrained on ImageNet and directly fine-tuned on DBT patches. Other studies fused information from different ROI patches within the same case. In Wang et al. (26), three multifaceted representations of the breast mass (gross mass, overview, and mass background) were extracted from the ROI and independently processed by a multi-scale multi-level features-enforced DenseNet.

Previous DBT classification work has primarily focused on classifying individual slices, which may not accurately represent the entire image-level structure of breast lesions. Some works attempt to crop whole images into different sections and sample neighboring sections as inputs (15, 17, 27). Predictions from different slices of the same case may yield conflicting results when processed independently by the model. Additionally, the breast lesion width varied in size from 10 mm to even more than 50 mm, which will harm the deep learning classification performance (28).

Another trend in recent studies is to focus on deeper neural network architectures to extract complex hierarchical features, while neglecting potentially valuable information captured by shallower neural network models (29). Some studies have compared the performance of deep and shallow networks in other breast image diagnosis systems. Kaya et al. (30) investigated this through an internal classifier network design, concluding from experimental results that deeper networks may induce overthinking phenomena that lead to computational resource waste that can be destructive for prediction tasks. The choice between these architectures is currently done by trial and error (31). Consequently, numerous experiments have attempted to incorporate shallow feature branches into deeper network architectures, which explicitly model and collaboratively optimize both shallow and deep features for tasks such as image classification and segmentation. For example, (32) proposed a Dual-Branch-UNet model for the vascular segmentation task, in which the deeper branches input low-resolution images and the shallower network branches process high-resolution images to extract spatial detail information. Gao et al. (33) proposed a novel Shallow-Deep CNN architecture for breast cancer diagnosis. In this architecture, a shallow network synthesizes contrast-enhanced-like images from standard mammograms, while a deep network analyzes lesion features in actual contrast-enhanced scans. These methods are inspired by the procedure performed by radiologists in which they combine different types of features (e.g., morphology and appearance) to perform diagnosis (34), which can potentially be extracted with different subnetworks.

Many medical imaging modalities only provide grayscale images, yet numerous studies have demonstrated that incorporating color information through colorization methods can significantly enhance various downstream tasks. These approaches operate under the hypothesis that pseudo-color enhancement algorithms (PCE), which transform grayscale images into pseudo-color representations, improve visual clarity and accentuate subtle details for both human interpretation and computational models, a premise substantiated by experimental results across multiple domains. El-Shazli et al. (15) applied PCE to the DBT grayscale images; adjustments in the hue, saturation, and value (HSV) color space, particularly the hue channel adjustments, were helpful to differentiate between different tissue regions. This approach has been assumed to avoid the coupling effect of simultaneously modifying color and brightness in RGB channels.

Additionally, Huang et al. (35) achieved superior segmentation accuracy in ultrasound tumor detection using PCE transformations. Zhang et al. (36) developed a specialized colorization network model that generates anatomically vivid tumor representations from PET-CT scans. Beyond conventional intensity-based color mapping, researchers like (37, 38) have pioneered multi-slice fusion techniques, where they composite different slice views of breast lesions into three-channel pseudo-RGB images, subsequently demonstrating measurable improvements in breast mass classification performance.

Inspired by the shallow deep neural network structure and PCE, to tackle the limitation of DBT diagnosis, we develop a PCE-based DBT Dual-Net model, which can effectively integrate both low and high-level features for various-sized breast lesions.

In summary, our key contributions are as follows:

We propose a DBT Dual-Net architecture that leverages features from both shallow and deep networks to improve discriminative capability while maintaining interpretability. To help the Dual-Net better extract a variety of features, we propose to use similarity loss to constrain the learned feature map.
We propose an HSV PCE method for DBT classification, which can improve the DBT classification performance.
We introduce an image-level classification framework that aggregates lesion information across multiple slices from the same patient, which provides a clinical application solution.

## 2 Methodology

**Figure 1:**
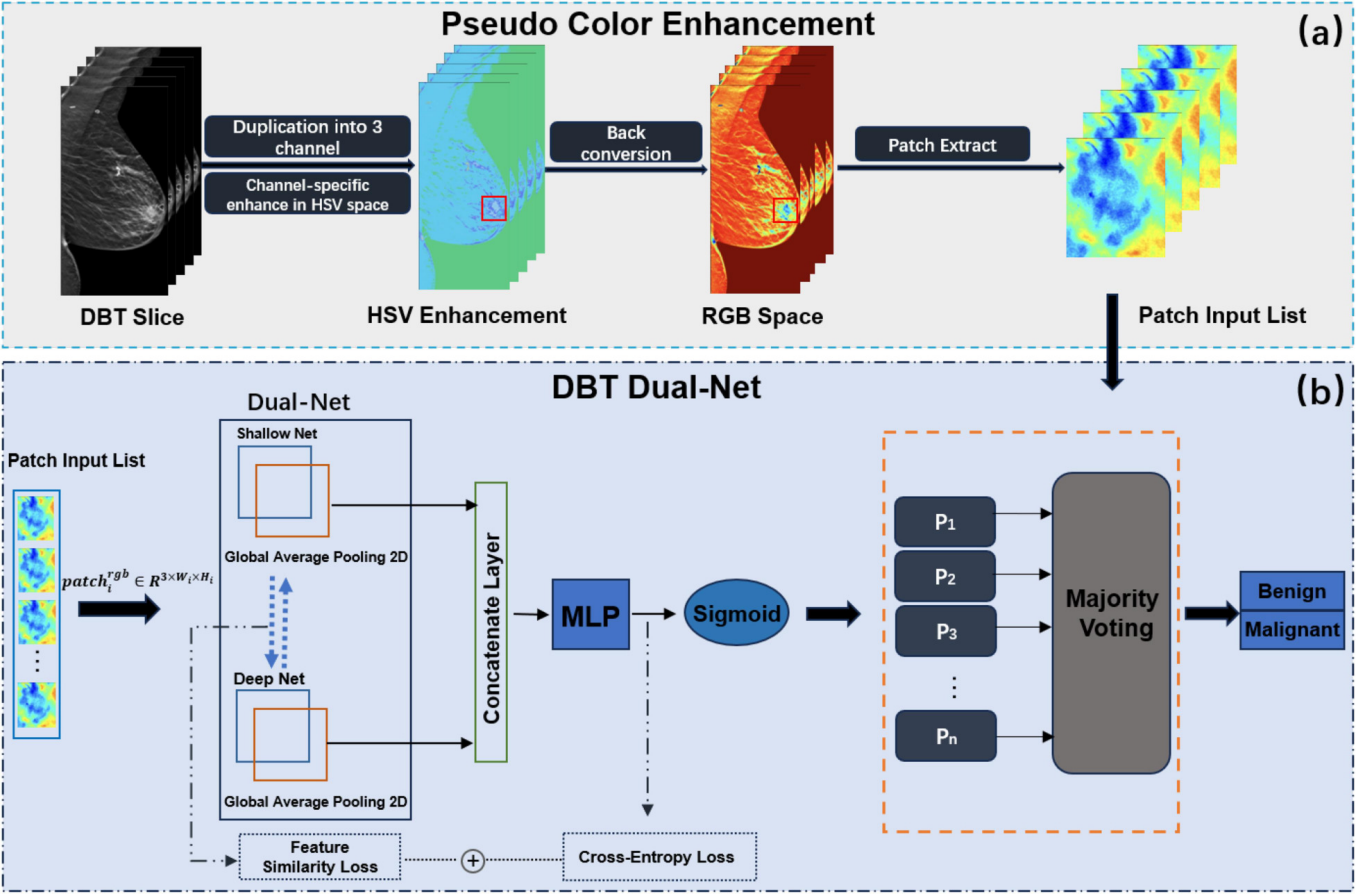
Overview of the proposed DBT classification framework. The framework incorporates two parts, **(a)** PCE and **(b)** DBT Dual-Net.

Figure 1 illustrates the proposed DBT lesion classification framework, which consists of two stages: PCE and the application of the Dual-Net model. First, pseudo-color patch augmentation was applied, where the original grayscale patches were transformed in the HSV color space to enhance discriminative features while preserving structural integrity. Second, a dual-branch classification network processed each augmented patch to extract both shallow and deep features. Based on the extracted features, the network predicted patch-wise probabilities for benign and malignant classification, which were aggregated into a final image-level prediction through a majority voting mechanism to ensure robustness against inter-slice variability.

### 2.1 Pseudo-color enhancement

Inspired by (15), we propose a pseudo-color patch enhancement method to improve the visual visibility of DBT lesion regions, as shown in Figure 1a. We applied a transformation based on the HSV color space and conducted comparative experiments to assess its impact on classification performance. The enhancement operation was to enhance hue, saturation, and brightness value.

After preprocessing, each input sample consists of n grayscale patches, denoted as ${\mathrm{patch}}_{i}\in R^{W\times H}$, where i=1,2,…,n. Each single-channel patch undergoes a three-step transformation: conversion to HSV, channel-specific enhancement and back to RGB.

The enhancement procedure modifies HSV channels as follows:

• **Hue adjustment:** A fixed shift ΔH is added to the hue channel and wrapped within the valid range [0,179] using modulo arithmetic, as shown in Equation 1:
  $$H_{\text{adjusted}}=(H+\Delta H)\;\mathrm{mod}\;\;180$$ (1)
• **Saturation scaling:** The saturation channel is scaled by a factor α, with values clipped to the range [0,255], as shown in Equation 2:
  $$S_{\text{adjusted}}=\min(S\times \alpha ,255)$$ (2)
• **Brightness scaling:** The value (brightness) channel is scaled by a factor β, also clipped to [0,255], as shown in Equation 3:
  $$V_{\text{adjusted}}=\min(V\times \beta ,255)$$ (3)

The adjusted channels were then merged to form the enhanced HSV image: $I_{\text{\textbackslash{},final}}=\text{HSV}(H_{\text{adjusted}},S_{\text{adjusted}},V_{\text{adjusted}})$ Finally, $I_{\text{\textbackslash{},final}}$ was converted back to RGB space to produce the enhanced image used in the classification pipeline.

**Figure 2:**
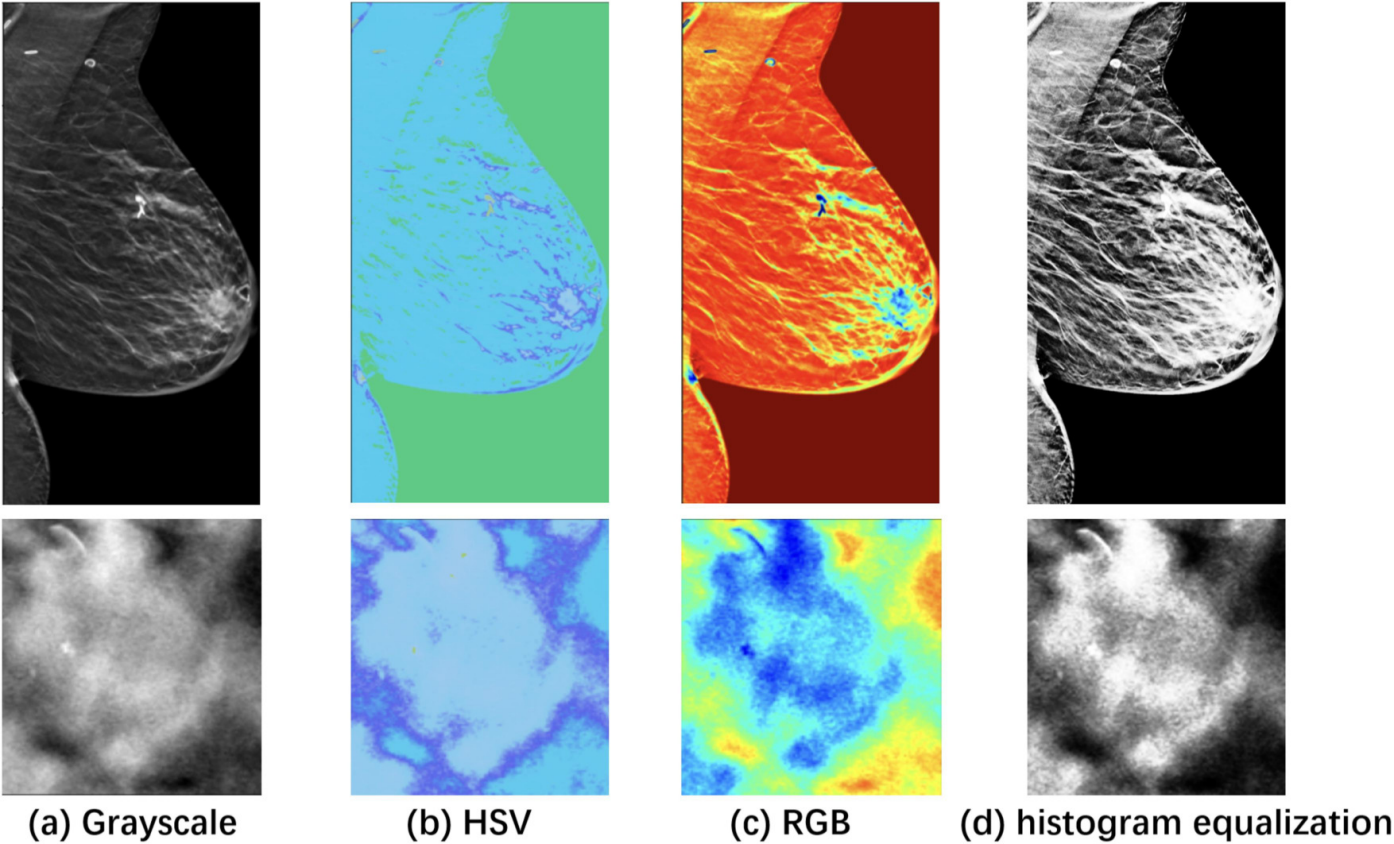
Visualization of a specific case with the ROI zoomed in. **(a)** Grayscale raw image; **(b)** Pseudo-color enhanced image in HSV; **(c)** Image transformed back to RGB after enhancement. **(d)** Raw image after histogram equalization, shown as a reference. As shown, image enhancement [columns **(c)** and **(d)**] makes features easier to identify compared to the raw image **(a)**.

**Figure 3:**
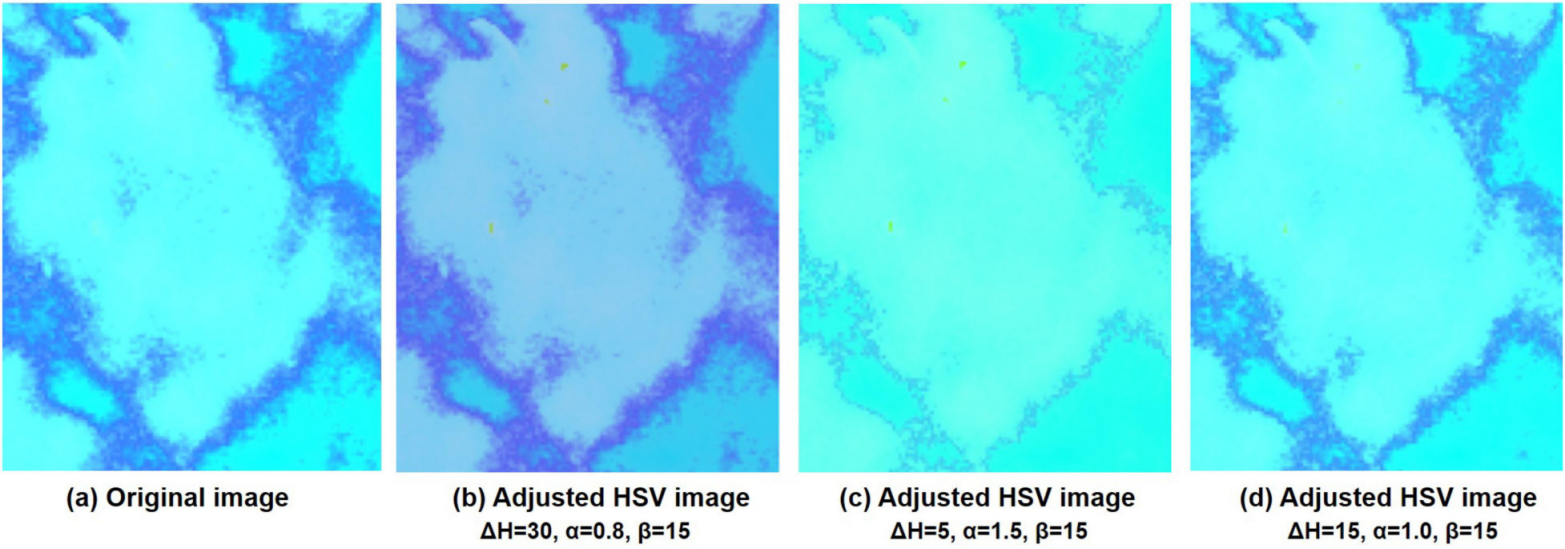
Comparison of the HSV images and after adjusting the different values of Hue, Saturation, and Value at different adjusted parameters. **(a)** is the original HSV converted image and **(b–d)** are adjusted HSV images at different parameter settings.

Figure 2 visualizes the differences between the original and pseudo color-enhanced patches. After mapping the grayscale image to color, compared to Figures 2a, b shows clearer irregular edges of the tumor, while Figure 2c exhibits blurred tumor edges but more prominent internal features. For comparison, the Figure 2d shows the image after histogram equalization. To find the optimal HSV adjustment parameter, Figure 3 highlights the impact of HSV-based adjustments on the lesion area and surrounding tissues. After HSV-based adjustment, the image enhances tumor boundary contrast while avoiding the excessive internal contrast seen in the original HSV image, which can lead to loss of subtle texture details. Based on this, the enhancement parameters were empirically set to ΔH=30, α=0.8, and β=15 in the following experiment.

### 2.2 DBT Dual-Net

The DBT Dual-Net which was designed to extract and fuse complementary features from both shallow and deep neural network branches for robust lesion classification. As shown in Figure 1b, the architecture consists of two parallel feature extractors: a shallow branch for capturing fine-grained spatial details, and a deep branch for extracting high-level semantic representations. Each input patch was featured independently by both branches. The overall Dual-Net formulation is, as shown in Equation 4:

$$F(x)=h\left([P_{s}(F_{s}(x));P_{d}(F_{d}(x))]\right).$$ (4)

Let X denote the space of preprocessed DBT lesion patches, where each cropped lesion slice x∈X with dimensions $R^{3\times h\times w}$. The proposed **Dual-Net** model F is designed as a composition of two parallel feature extractors and a fusion-based classifier:

**Shallow branch:** $F_{s}\;:\;X\to R^{d_{s}}$, a shallow neural network that extracts low-level spatial features.The $d_{s}$ denotes as size of the extracted shallow feature vector.
**Deep branch:** $F_{d}\;:\;X\to R^{d_{d}}$, a deep neural network that extracts high-level semantic features. The $d_{d}$ denotes as size of the extracted deep feature vector
**Feature fusion:** The outputs are projected to a common space via linear mappings $P_{s}\;:\;R^{d_{s}}\to R^{d}$ and $P_{d}\;:\;R^{d_{d}}\to R^{d}$, then concatenated:
  $$z=[P_{s}(F_{s}(x));P_{d}(F_{d}(x))]\in R^{2d}.$$
**Classifier:** $h\;:\;R^{2d}\to [0,1]$, a fully connected layer with sigmoid activation for binary classification.

#### 2.2.1 Dual-branch feature extraction

The dual-branch included a shallow network branch $F_{s}$ and a deep network branch $F_{d}$. They were combined, and the backbone of their feature extraction parts was retained. The categorization of networks into shallow or deep architectures was based on parameter counts and layer depths, with specific classification criteria presented in Table 1.

**Table 1:** The shallow and deep network for Dual-Net.

Category	Model	Layers	Params(M)
	MobileNetV2	17	3.4
Shallow	AlexNet	8	60
	ResNet18	18	11
	ResNet50	50	25
Deep	ResNeXt50	50	25
	DenseNet121	121	8
	DenseNet201	201	20

The shallow and deep branches are distinguished based on the parameter and the number of layers, summarized in Table 1. Shallow networks (≤50 layers), such as AlexNet and ResNet18, capture fine anatomical details, whereas deep networks (>50 layers), such as ResNet50, ResNeXt50, DenseNet121 and DenseNet201, excel at extracting high-level semantic features.

The deep branch outputs a global feature vector $f_{d}\in R^{d_{d}\times h_{d}\times w_{d}}$, while the shallow branch outputs a spatial feature map $f_{s}\in R^{d_{s}\times h_{s}\times w_{s}}$. An adaptive pooling operation Pool(⋅) is applied to unify spatial dimensions, followed by a flattening operation v=Flatten(Pool(f)). The vectors $v_{d}$ and $v_{s}$ are projected into a shared d-dimensional space via $\hat{\;f}=P(v)=Wv+b$ respectively. Then, the projected features $\left({\hat{\;f}}_{d},{\hat{\;f}}_{s}\right)$ are used for the similarity loss to encourage complementary representations. The original features $\left(v_{d},v_{s}\right)$ are concatenated to form: $z=[v_{d},v_{s}{]}^{T}\in R^{d_{d}+d_{s}},$ which is passed to the classifier.

#### 2.2.2 Image-level prediction

**Majority voting**—For an image consisting of n patches $\{x_{i}{\}}_{i=1}^{n}$, patch-level predictions $F(x_{i})\in [0,1]$ are aggregated into an image-level label $\hat{y}$ via majority voting, as shown in Equation 5:

$$\hat{y}=\mathrm{mode}\;\left(\{1[F(x_{i})\geq 0.5]{\}}_{i=1}^{n}\right).$$ (5)

This aggregation mitigates inter-slice variability and leverages the spatial heterogeneity of lesion appearance in DBT volumes.

**Average voting**—As a comparison, this approach computes the mean predicted probability across all n patches in a case, then applies a threshold of 0.5 to determine the final case label, as shown in Equation 6:

$$\hat{y}=\left\{ \begin{array}{cc} 1, & \text{if}\;\frac{1}{n}\sum_{i=1}^{n}p_{i}\geq 0.5,\\ 0, & \text{otherwise.} \end{array}\right.$$ (6)

where $p_{i}$ is the predicted slice-level probability score.

#### 2.2.3 Loss function

To help the two branches to capture representations efficiently, we employed a joint loss that combined the standard binary cross-entropy (BCE) classification loss with a similarity loss between the projected features of the two branches. The total loss was defined as a weighted sum of these components and averaged across all slices in the batch.

For each input slice $x_{i}$, the model produced a predicted probability ${\hat{y}}^{\left(i\right)}$. The BCE loss for slice i is computed as shown in Equation 7:

$$L_{\text{BCE}}^{\left(i\right)}=-\left(y^{\left(i\right)}\log\;({\hat{y}}^{\left(i\right)})+(1-y^{\left(i\right)})\log\;(1-{\hat{y}}^{\left(i\right)})\right),$$ (7)

where $y^{\left(i\right)}$ denotes the ground truth label.

In addition, to encourage the dual branches to extract feature representations that are, to some extent, consistent. For this, we introduce a similarity loss between the projected features ${\hat{\;f_{s}}}^{\left(i\right)}$ and ${\hat{\;f_{d}}}^{\left(i\right)}$ of slice i. This loss was based on mean squared error, as shown in Equation 8:

$$L_{\text{Sim}}^{\left(i\right)}={\left({\hat{\;f_{s}}}^{\left(i\right)}-{\hat{\;f_{d}}}^{\left(i\right)}\right)}^{2},$$ (8)

where ${\hat{\;f_{s}}}^{\left(i\right)}$ and ${\hat{\;f_{d}}}^{\left(i\right)}$ are the outputs of linear projections applied to the features from each branch.

Finally, the overall training objective was formulated as shown in Equation 9:

$$L=\frac{1}{N}\sum_{i=1}^{N}\left(L_{\text{BCE}}^{\left(i\right)}+\lambda \cdot L_{\text{Sim}}^{\left(i\right)}\right),$$ (9)

where N is the number of slices in the batch and λ is a balancing coefficient that controls the contribution of the similarity term.

### 2.3 Dataset

For this study, we utilized the public dataset from the Breast Cancer Screening-Digital Breast Tomosynthesis (BCS-DBT) dataset, publicly available in the Cancer Imaging Archive (39). This dataset was obtained from Duke Health and made available in anonymized DICOM format. It consists of 22,032 breast tomosynthesis scans from 5,060 patients. The spatial resolution is 0.085 mm in the *XY* direction and 1 mm in the *Z* direction. The corresponding label files of the dataset contain annotations made by radiologists for each case, including the patient ID, the type of lesion (actionable, benign, and malignant), the imaging view associated with the case, the specific center slice number and position (bounding box) of the lesion. This dataset splits training, validation, and testing sets at the patient level. The distribution of malignant and benign cases, which are the ones used in this study, is shown in Table 2.

**Table 2:** Distribution of malignant and benign cases in DBT-BSC dataset.

Dataset	Malignant	Benign
Training	87	137
Validation	37	38
Test	64	69

**Figure 4:**
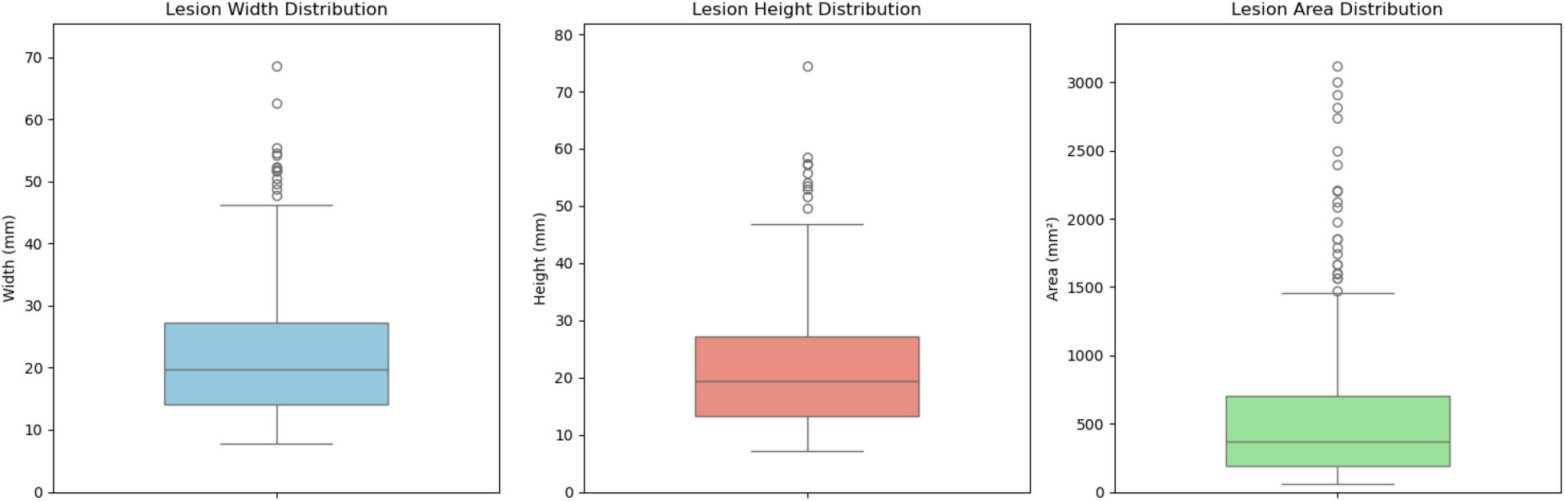
Comparative distribution of lesion physical dimensions: width (mm), height (mm), and area (${\mathrm{mm}}^{2}$).

**Figure 5:**
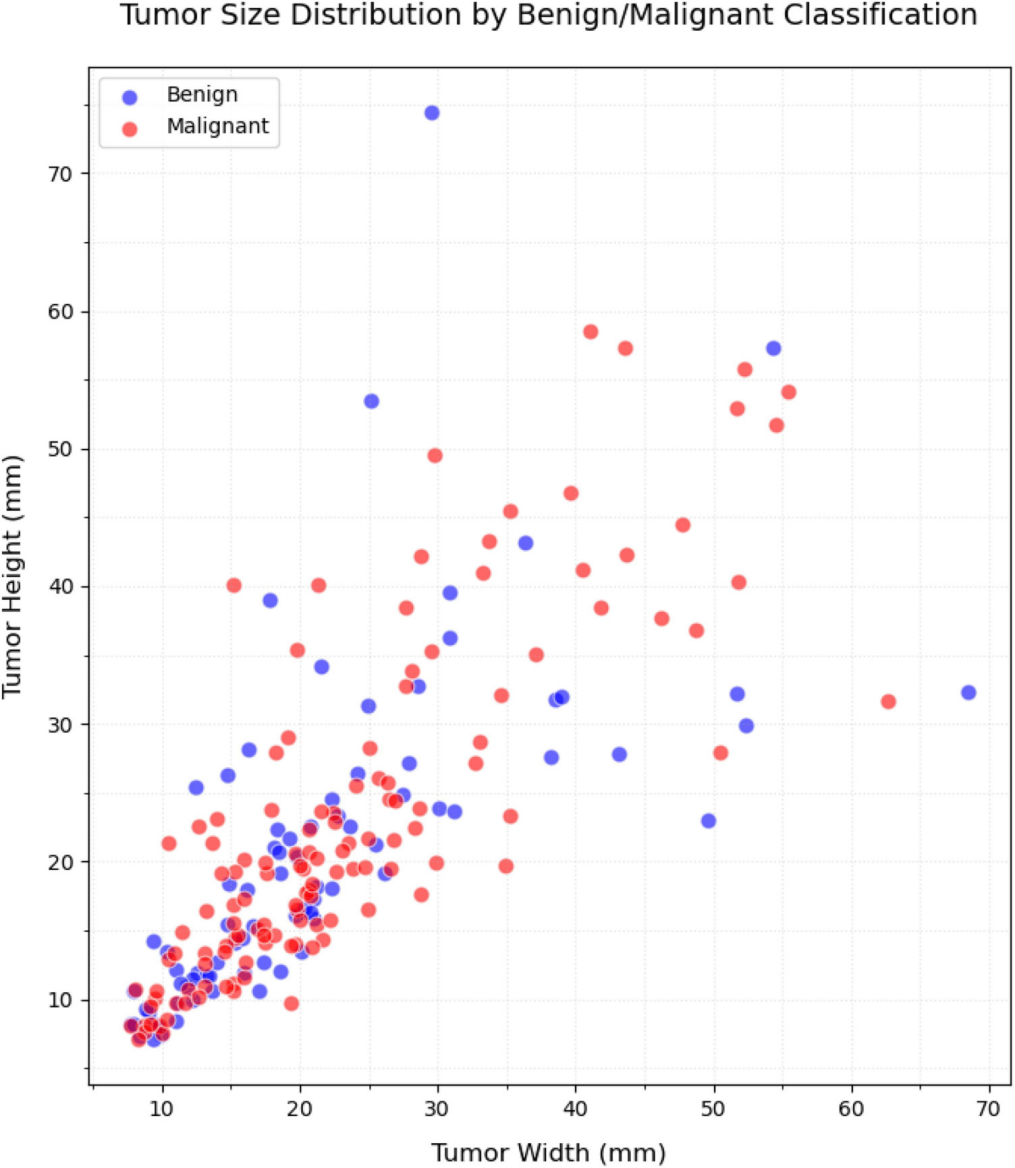
Distribution of the size (width/height) in lesion dimensions.

**Figure 6:**
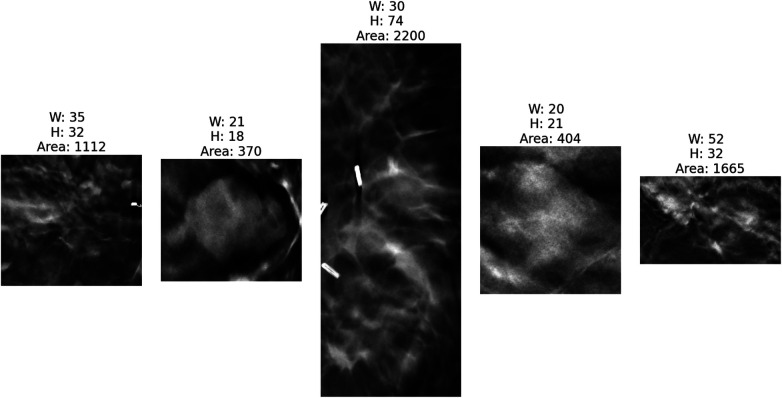
Five cropped lesions based on the annotated bounding boxes by radiologists with corresponding dimensional measurements (width, height, area) to illustrate size variability across the dataset. The bright regions on the first and third images are breast clips, which are used to mark the biopsy region. As shown, different lesions have different shapes and sizes.

Figures 4, 5 present the distribution of lesion width, height, and area in the training set, highlighting the considerable variability in lesion size. The lesions range from approximately 5 mm to 80 mm in width and height, with areas spanning from about 100 ${\mathrm{mm}}^{2}$ to over 3,000 ${\mathrm{mm}}^{2}$. Such large variations in lesion size may negatively impact the performance of deep learning–based diagnostic models. Figure 6 presents some selected examples for visual comparison. We preprocessed the images as follows:

#### 2.3.1 Lesion ROI extraction

Since DBT data contains images acquired from different views, all images were first uniformly flipped to a left-oriented alignment to standardize the dataset. For lesion region extraction, our processing pipeline implemented multi-axis standardization. In the XY-plane, lesion regions were extracted using annotated bounding box coordinates, with each box’s width and height expanded by 10 pixels in all directions (a total of 20-pixel expansion per dimension) to preserve the surrounding parenchymal context. We analyzed lesion thickness in every slice. We found that the smallest tumor had 10 mm diameter. By assuming lesions are roughly spherical, we selected 10 slices, equivalent to 10 mm, along the *Z*-axis around the slice that contained the annotation. In this way, we make sure that all considered slices contain tumoral tissue. Besides, the ROI intensities were normalized to the range [0,1], and the cropped regions were resized to 224×224 pixels.

#### 2.3.2 Patch sampling

**Figure 7:**
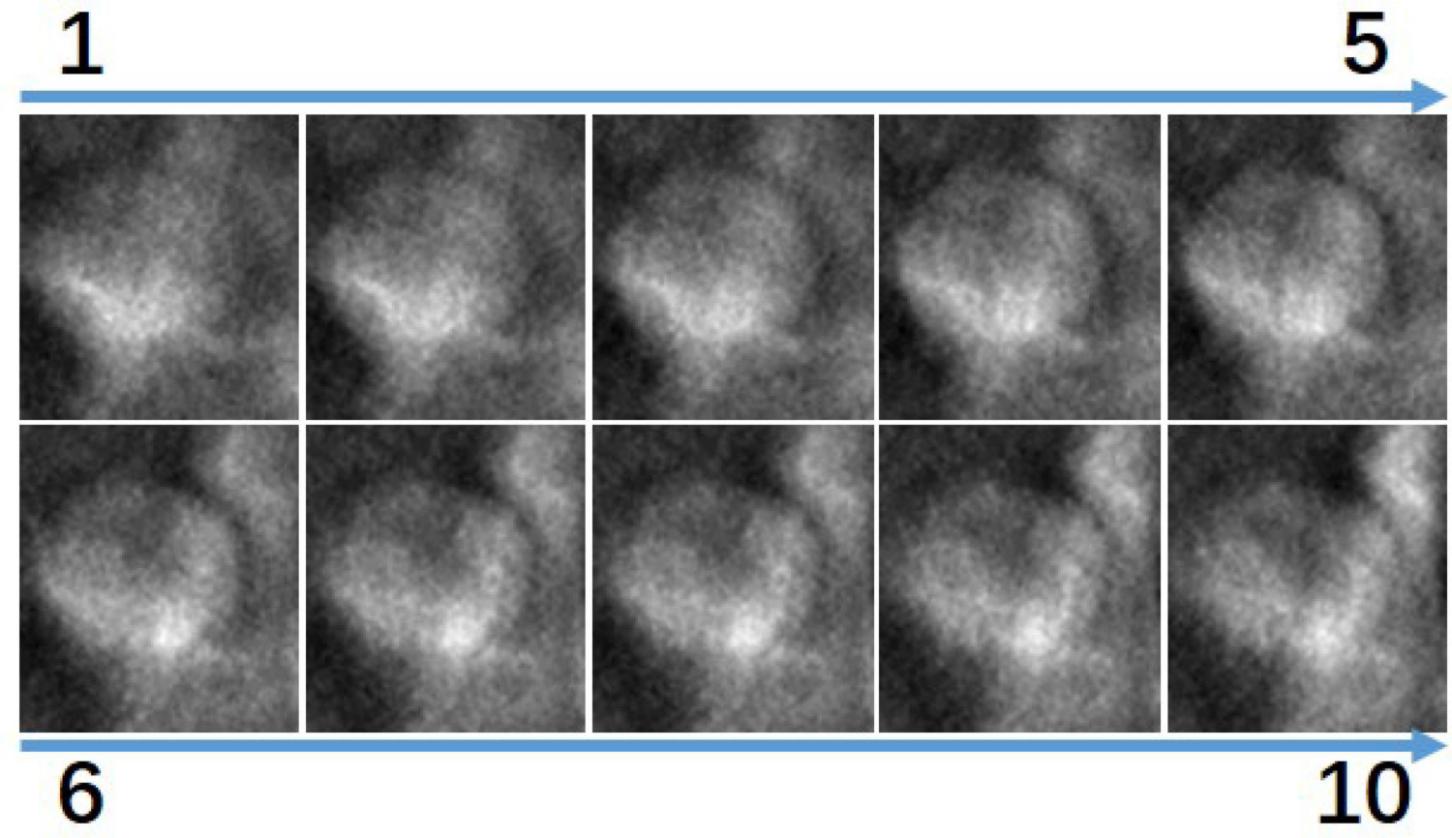
Visualization of the 10 slices in the *z* direction surrounding the smallest lesion in the dataset, ordered from superior to inferior. All images show lesion tissue.

After lesion extraction, for each DBT lesion case, a minimum 10 mm was initially available across the *Z*-direction. We employed a center-slice-based symmetrical sampling approach: we extracted different slices at symmetric positions around the lesion center. Figure 7 visualizes the smallest lesion case. As shown, all slices contain tissue with the lesion. To evaluate how different numbers of slice sampling affect diagnostic accuracy, we conducted controlled experiments in which both the training and validation phases used identical slice quantities per case.

#### 2.3.3 Data augmentation

Given the limited dataset size, augmentation techniques were applied to improve model generalization:

**Rotation**: Random rotation by $10^{\circ }$ to $30^{\circ }$ to improve rotational invariance.
**Translation**: Horizontal and vertical shifts to enhance robustness against off-center lesions.

## 3 Experimental results

### 3.1 Experimental setting

All models were developed using PyTorch and trained on NVIDIA A100 GPUs. We employed the Adam optimizer. The choice of learning rates was based on empirical selection within the range of $1\times 10^{-5}$ to $1\times 10^{-2}$ with a 10 times increase. The learning rate of $3\times 10^{-4}$ was the best. The training process consisted of 150 epochs for complete model convergence.

### 3.2 Performance measure

We evaluate classifiers using standard metrics: Accuracy (overall correctness), area under the ROC curve AUC, precision (correctness among predicted positives), sensitivity (fraction of actual positives detected), specificity (fraction of actual negatives correctly identified), and F1 (harmonic mean of precision and recall).

### 3.3 Results

First, we performed a sensitivity analysis on the different parameter settings of the proposed method. Finally, we compared the proposed method with competing classification methods.

#### 3.3.1 Parameter selection

##### 3.3.1.1 Combinations of different shallow and deep networks

**Figure 8:**
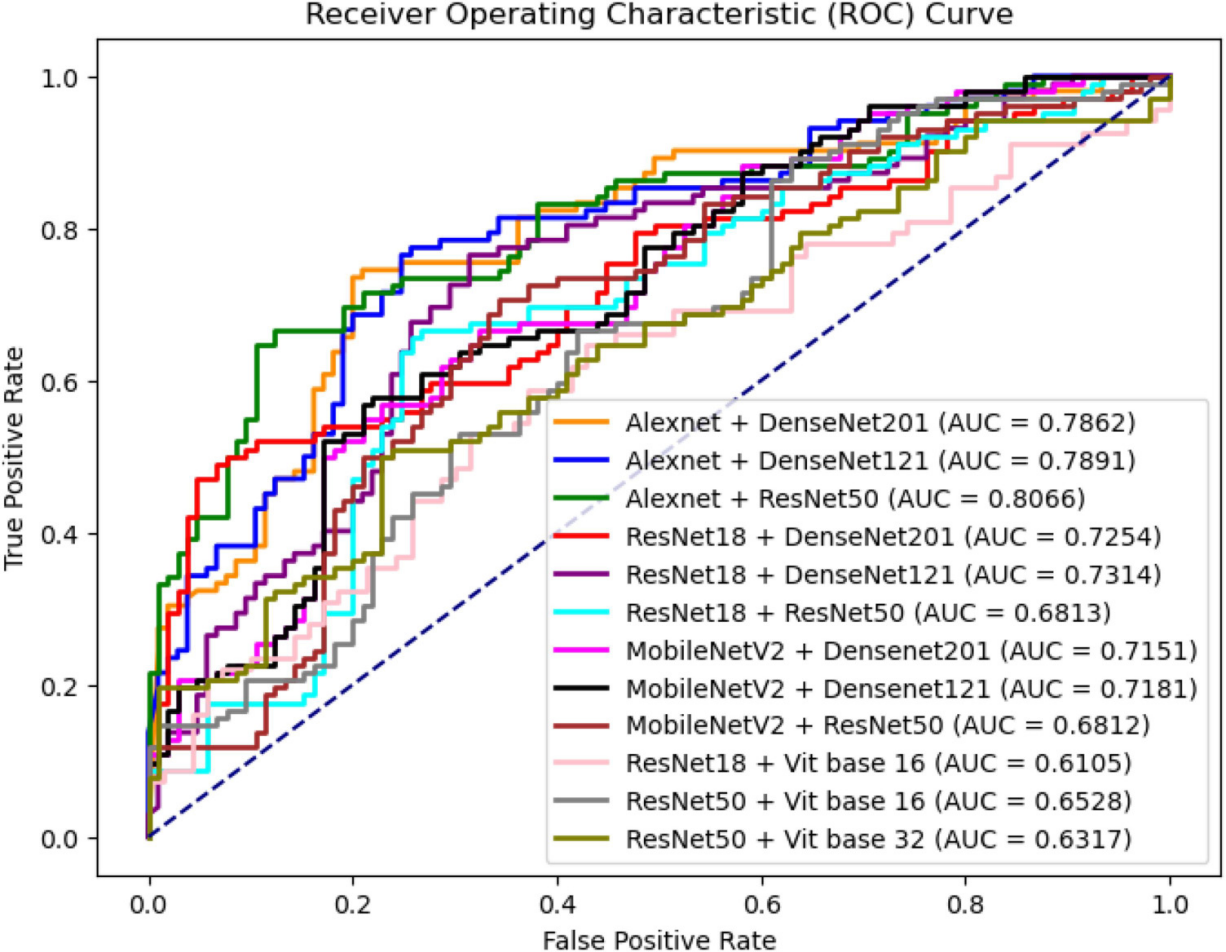
ROC curves of different combinations between shallow network and deep network architectures.

Table 3 shows the performance of the different combinations of shallow and deep networks in Dual-Net. Figure 8 also shows the ROC curves of the different combinations. As shown, except for precision and specificity, the combination of AlexNet and ResNet50 gave the best results. For this reason, this combination was used in the comparison with other methods. It is interesting to see that pure CNN-based combinations outperform hybrid methods that include ViTs.

**Table 3:** Performance comparison of different combination dual-branch models on DBT lesion classification at image level.

Model	Acc	AUC	Prec	Sens	Spec	F1
Alex + Dense201	70.16 ± 3.27	76.32 ± 6.86	66.74 ± 8.23	71.21 ± 5.66	61.68 ± 10.11	**74.16 ± 9.81**
Alex + Dense121	71.01 ± 5.98	71.01 ± 6.13	67.07 ± 7.21	78.21 ± 6.17	61.90 ± 5.72	73.3 ± 6.65
Alex + Res50	**75.36 ± 4.49**	**78.28 ± 5.70**	73.35 ± 4.73	**88.24 ± 4.52**	55.94 ± 13.50	72.73 ± 6.21
Res18 + Dense201	67.57 ± 4.82	71.24 ± 5.33	68.87 ± 9.17	68.31 ± 12.33	**75.19 ± 7.23**	67.69 ± 11.32
Res18 + Dense121	66.67 ± 4.76	71.21 ± 3.86	**74.76 ± 8.56**	71.94 ± 17.04	56.81 ± 29.47	71.44 ± 7.14
Res18 + ResNet50	64.32 ± 6.21	64.13 ± 5.45	69.58 ± 9.65	69.69 ± 12.34	68.27 ± 21.11	66.32 ± 9.33
Mobile + Dense201	68.12 ± 5.34	69.51 ± 6.12	63.64 ± 7.21	82.35 ± 6.17	54.29 ± 5.72	71.79 ± 6.65
Mobile + Dense121	68.84 ± 4.73	69.31 ± 6.34	67.43 ± 5.81	72.39 ± 10.11	54.86 ± 11.32	73.14 ± 8.17
Mobile + Res50	67.95 ± 3.23	65.48 ± 2.50	69.08 ± 12.37	73.57 ± 19.02	45.24 ± 29.67	68.51 ± 6.41
Res18 + ViT16	56.37 ± 5.21	58.25 ± 6.77	56.89 ± 11.2	64.23 ± 17.26	65.27 ± 17.21	61.57 ± 9.11
Res50 + ViT16	62.17 ± 3.67	66.83 ± 4.73	64.73 ± 9.43	67.82 ± 8.73	64.81 ± 12.36	67.40 ± 7.91
Res50 + ViT32	63.39 ± 4.17	62.71 ± 6.27	65.29 ± 8.49	70.29 ± 8.91	64.23 ± 11.23	64.57 ± 5.71

The bold values indicate the best performance.

##### 3.3.1.2 Weight factor at similarity loss

Concerning the hyperparameter λ of the similarity loss, we tested the performance of Dual-Net with different values. The λ was designed to constrain similarity between two features extracted from each branch. The result is shown in Table 4, which shows that λ=0.3 has the best performance for AUC, which is usually the preferred metric in clinical settings. Disregarding the similarity constraint also gives competitive results.

**Table 4:** Performance of Dual-Net with differnet λ values set for similarity loss.

λ	Acc	AUC	Prec	Sens	Spec	F1
0	**75.36**	78.28	**73.35**	**88.24**	55.94	72.73
0.1	72.46	78.81	69.74	77.94	**67.14**	73.61
0.3	74.48	**82.18**	64.44	85.29	54.29	73.42
0.5	70.29	74.07	62.35	77.94	54.29	69.28

The bold values indicate the best performance.

##### 3.3.1.3 Effect of pseudo-color enhancement

To validate the effectiveness of PCE, we compare the classification performance without image enhancement, with PCE, and with histogram equalization. As shown in Table 5, incorporating PCE yields the best performance, while histogram equalization also improves performance but to a lesser extent. Specifically, accuracy increased from 65.94% to 75.36%, and AUC rose from 69.45 to 77.80, indicating better overall discrimination. Precision, specificity, and F1-score also improved substantially, reflecting more balanced performance across classes. We compare to the without and the best lambda weight factor with the similarity loss.

**Table 5:** Classification performance using different image enhancement methods.

Method	Acc	AUC	Prec	Sen	Spec	F1
Without enhancement	65.94	69.45	61.80	80.88	51.43	70.06
Histogram equalization	66.67	74.43	60.47	76.47	51.43	67.53
PCE	**74.48**	**82.18**	**64.44**	**85.29**	**54.29**	**73.42**

The bold values indicate the best performance.

##### 3.3.1.4 Different numbers of adjacent slices

We also compared the classification performance at the image level with different numbers of adjacent slices as input. We choose m=1,2,3 and ultimately form a total of 2m+1 slices to predict the image-level results, which serve as the input. The results obtained from voting with different numbers of patch inputs, using the previously best Dual-Net model for this evaluation shown in Table 6. The results show that m=1 acquired the optimal classification performance (i.e., the center slice plus the adjacent slices), but incorporating excessive slice information does not necessarily improve tumor characterization performance.

**Table 6:** Comparison of different numbers of adjacent patch inputs at image level.

Number	Acc	AUC	Prec	Sens	Spec	F1
1	**74.48**	**82.18**	64.44	**85.29**	54.29	**73.42**
2	73.91	75.44	**70.59**	70.59	**71.43**	70.59
3	70.29	73.00	64.29	66.18	64.29	65.22

The bold values indicate the best performance.

#### 3.3.2 Comparison with previous methods

To demonstrate superiority of the proposed method, we compared the proposed method with different fine-tuned deep learning classifiers, including ResNet (40), AlexNet (41), ResNeXt50 (42), MobileNetV2 (43), DenseNet (44), ViT (45) with pretrained weights. These classifiers were selected as they cover architectures with different depths and complexities. Moreover, a feature pyramid network (FPN) (46) with ResNet50 backbone was also employed for performance comparison. This architecture integrates feature maps from four different convolution layers, which are subsequently concatenated before making final predictions. We performed a five-fold cross-validation in the experiments.

##### 3.3.2.1 Classification performance at slice level

**Figure 9:**
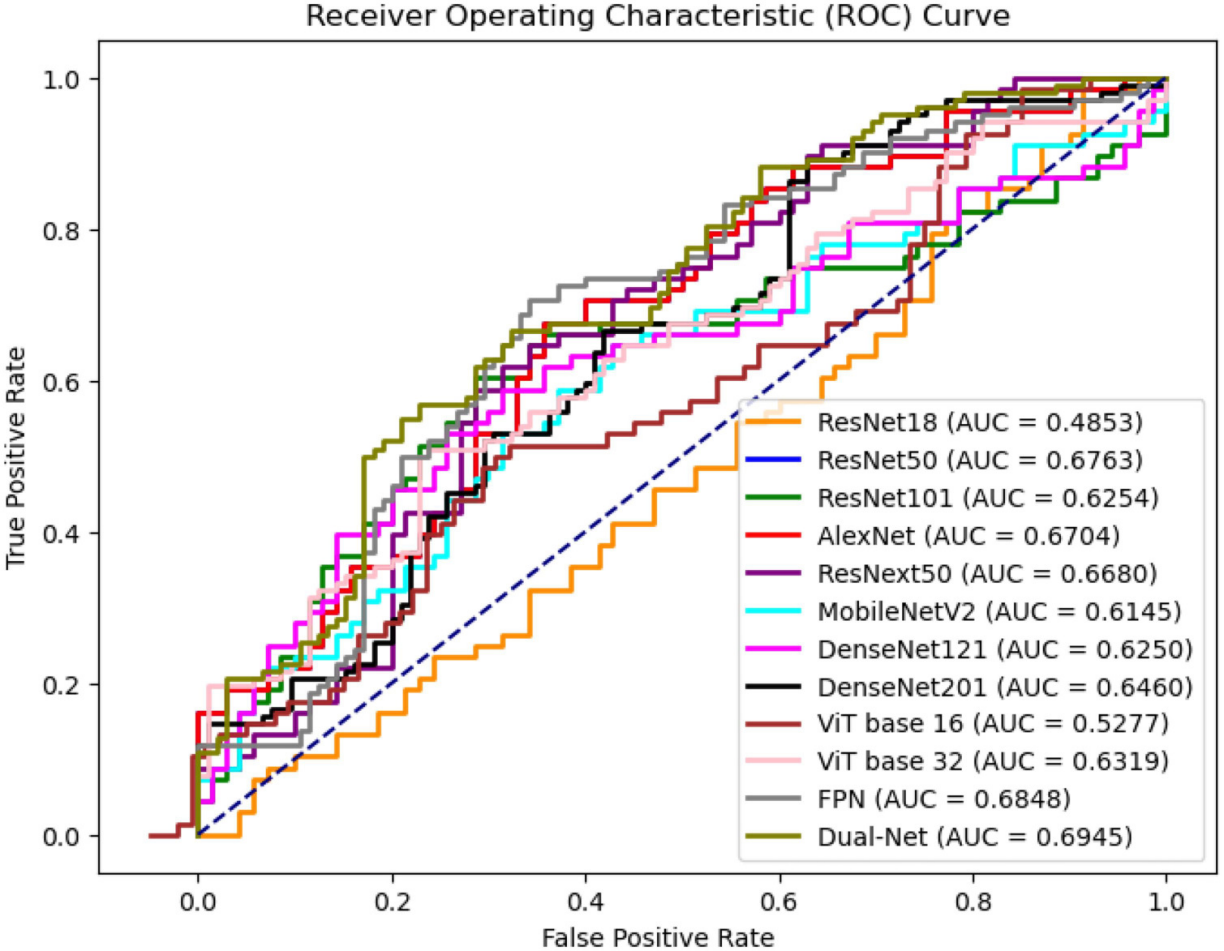
ROC curves of slice-level prediction at different models at test set.

Table 7 compares the performance of different network architectures for DBT lesion classification, and Figure 9 presents the ROC curves of the corresponding models. The results do not clearly that either shallow or deep networks are better for DBT classification However, our proposed method (Dual-Net) results have the best performance.

**Table 7:** Comparison of the proposed method with different classification methods at the slice level.

Model	Acc	AUC	Prec	Sen	Spec	F1
ResNet18	55.24 ± 3.25	48.53 ± 5.94	52.00 ± 15.42	52.00 ± 13.24	54.84 ± 18.94	58.24 ± 12.33
ResNet50	65.24 ± 1.08	64.94 ± 2.31	59.52 ± 7.42	61.54 ± 10.94	56.25 ± 9.24	65.31 ± 14.36
ResNet101	61.59 ± 2.21	62.54 ± 4.13	56.88 ± 11.42	91.18 ± 4.21	32.86 ± 14.19	68.06 ± 13.35
AlexNet	53.70 ± 9.33	65.68 ± 6.97	54.76 ± 19.90	55.00 ± 30.00	45.00 ± 40.00	53.00 ± 28.31
ResNeXt50	57.74 ± 1.03	64.38 ± 4.25	68.60 ± 7.28	**95.59 ± 1.34**	17.14 ± 13.43	68.06 ± 5.57
MobileNetV2	59.52 ± 6.03	61.56 ± 2.82	61.54 ± 7.60	69.57 ± 16.32	47.37 ± 23.44	65.31 ± 13.61
DenseNet121	60.14 ± 3.74	62.50 ± 4.04	62.00 ± 7.44	65.00 ± 13.11	55.00 ± 18.42	63.00 ± 9.28
DenseNet201	57.74 ± 1.03	64.38 ± 4.25	**68.60 ± 7.28**	58.19 ± 9.99	56.86 ± 13.43	62.13 ± 5.57
ViT Base-16	52.17 ± 7.88	52.77 ± 6.54	66.25 ± 8.10	87.10 ± 10.66	28.03 ± 23.70	**74.68 ± 6.65**
ViT Base-32	60.95 ± 6.84	63.76 ± 3.66	63.32 ± 6.84	87.26 ± 12.11	18.62 ± 23.61	72.26 ± 7.24
FPN	61.84 ± 4.56	62.81 ± 5.77	62.64 ± 11.37	55.88 ± 14.63	**67.62 ± 12.43**	59.07 ± 7.45
**Dual-Net**	**65.94 ± 5.70**	**69.45 ± 4.73**	61.80 ± 9.11	80.88 ± 10.31	51.43 ± 18.27	70.06 ± 7.33

The measurements are given as mean ± standard deviation on a 5-fold cross-validation setting.

The bold values indicate the best performance.

**Figure 10:**
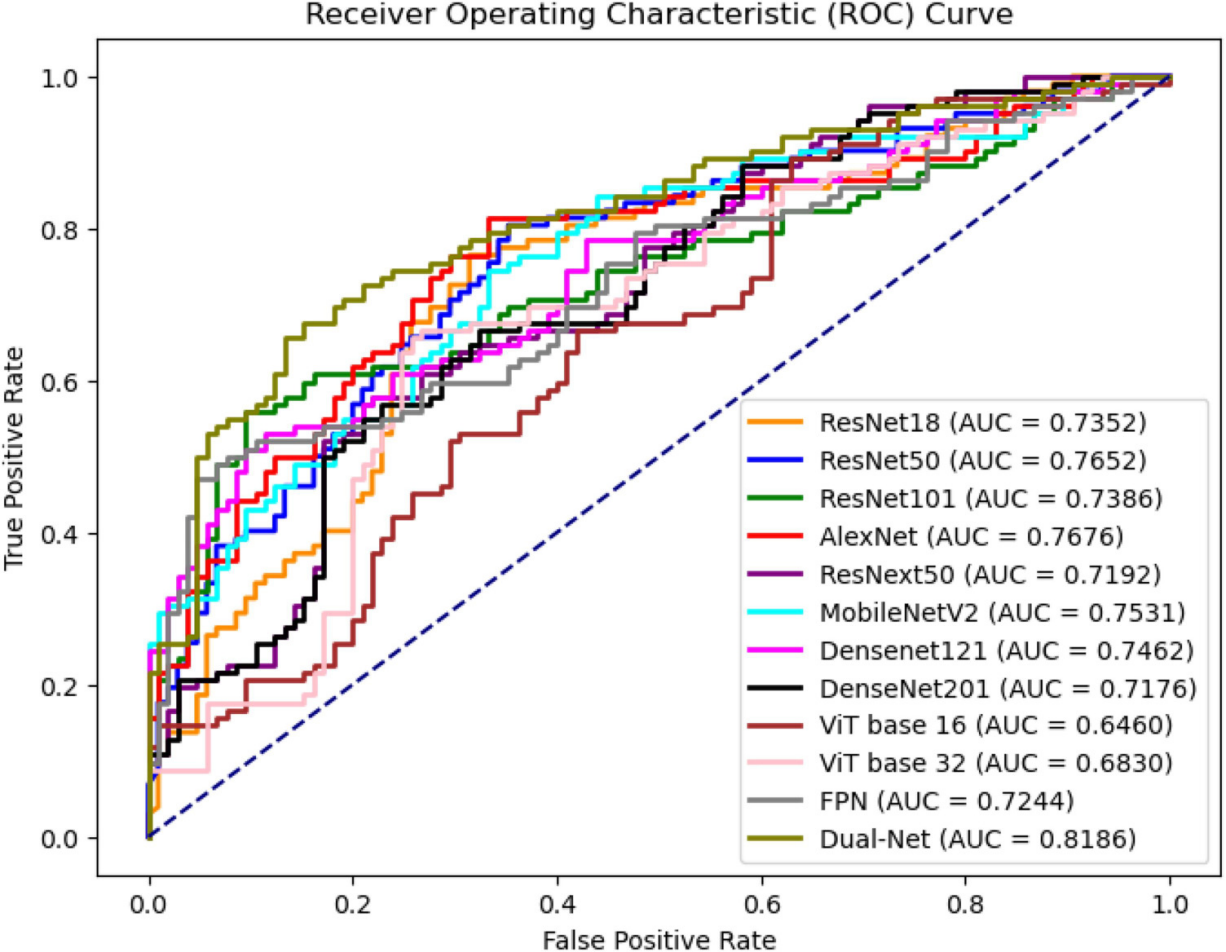
ROC curves of image level prediction with majority voting at test set.

##### 3.3.2.2 Classification performance at image level

To obtain image-level predictions from DBT volumes, a voting-based aggregation strategy was applied. Table 8 and Figure 10 report the corresponding image-level classification performance. Each image contains n patches that share a single ground-truth label. Two aggregation methods were evaluated: majority voting (${\mathrm{Acc}}_{1}$) and average voting (${\mathrm{Acc}}_{2}$) denoted in Table 8. For AUC and other threshold-independent metrics, the image-level predicted probabilities are from the averaged selected patches. As shown in Table 8, except for precision, Dual-Net was the best method. The majority voting strategy focuses on the consensus among patch-level predictions, whereas average voting incorporates the confidence of each patch prediction into the aggregation process.

**Table 8:** Comparison of the proposed method with different classification methods at the image level. Acc1 and Acc2 refer to as majority and average voting, respectively.

Models	Acc1	Acc2	AUC	Prec	Sen	Spec	F1
ResNet18	71.29 ± 5.43	68.42 ± 3.29	72.39 ± 3.64	70.82 ± 7.10	71.75 ± 7.99	54.32 ± 7.47	70.79 ± 4.48
ResNet50	70.67 ± 2.13	71.01 ± 1.37	72.65 ± 1.97	**72.02 ± 6.97**	77.87 ± 16.34	50.50 ± 18.56	73.22 ± 4.95
ResNet101	65.48 ± 5.34	64.17 ± 2.07	74.71 ± 3.27	67.69 ± 9.73	70.92 ± 20.52	45.03 ± 23.77	65.83 ± 8.23
AlexNet	67.64 ± 4.42	67.06 ± 5.78	67.70 ± 6.48	69.81 ± 4.18	79.81 ± 6.98	37.25 ± 14.75	72.43 ± 3.40
ResNeXt50	66.67 ± 6.63	64.47 ± 4.75	71.97 ± 3.13	70.50 ± 15.07	75.68 ± 11.26	40.87 ± 33.35	64.90 ± 18.64
MobileNetV2	71.01 ± 3.57	65.72 ± 5.17	72.81 ± 4.37	68.32 ± 8.59	85.04 ± 7.81	38.30 ± 15.78	74.95 ± 2.53
DenseNet121	71.26 ± 2.23	68.70 ± 3.32	69.70 ± 4.19	69.67 ± 9.72	74.99 ± 14.53	47.57 ± 23.28	70.56 ± 3.76
DenseNet201	71.01 ± 1.56	67.46 ± 2.13	70.39 ± 1.46	68.10 ± 7.68	79.84 ± 8.21	41.83 ± 11.45	72.67 ± 0.85
ViT Base-16	65.71 ± 7.00	62.47 ± 4.37	63.81 ± 5.20	71.24 ± 7.09	71.52 ± 10.12	**56.10 ± 3.70**	71.14 ± 7.75
ViT Base-32	67.62 ± 4.15	62.32 ± 3.41	67.79 ± 3.59	69.76 ± 7.51	83.91 ± 6.53	43.69 ± 10.06	**75.72 ± 3.81**
FPN	67.79 ± 2.77	67.28 ± 3.24	71.68 ± 4.61	51.16 ± 10.24	87.48 ± 6.73	27.54 ± 18.63	67.01 ± 8.34
**Dual-Net**	**74.48 ± 1.18**	**71.57 ± 3.22**	**82.18 ± 3.62**	64.44 ± 4.57	**85.29 ± 6.31**	54.29 ± 14.62	73.42 ± 3.32

The measurements are given as mean ± standard deviation on a 5-fold cross-validation setting.

The bold values indicate the best performance.

##### 3.3.2.3 Statistical analysis

To validate the performance improvements, the final version of Dual-Net was used to compare with the best-performing slice-level and image-level models in terms of AUC. A paired per-case analysis was conducted, and statistical significance was assessed via McNemar tests with 2 × 2 contingency tables. Table 9 shows that Dual-Net significantly outperforms the best baselines (p<0.05).

**Table 9:** Statistical significance analysis for comparison between Dual-Net with the best performing baseline models at slice and image levels.

Models	$\chi^{2}$	*p*-value
AlexNet (Slice-level)	27.04	$2.00\times 10^{-7}$
ResNet101 (Image-level)	24.03	$9.4\times 10^{-6}$

All comparisons yielded statistically significant differences (p<0.05), confirming the robustness of our final Dual-Net architecture and its superiority across diverse configurations and preprocessing approaches.

#### 3.3.3 Visualization results

##### 3.3.3.1 t-SNE Visualization

**Figure 11:**
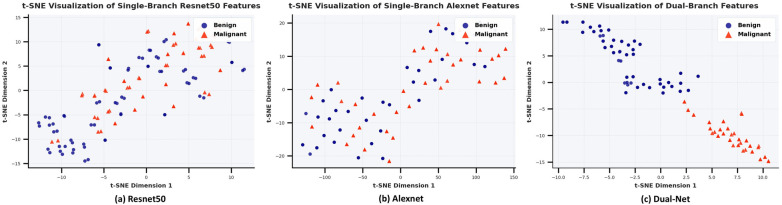
Comparative t-SNE analysis of learned feature representations: **(a)** ResNet50 single-branch, **(b)** Alexnet single-branch, **(c)** Dual-net. The distributions in two colors represent the clustering of benign (blue) and malignant (red) samples in the 2D feature space after t-SNE dimensionality reduction of the features extracted by the model, where the dual-branch network demonstrates superior inter-class separation and intra-class compactness.

To investigate why Dual-Net outperforms the single-branch network in classification, t-distributed Stochastic Neighbor Embedding (t-SNE) (47) is used. We separately extracted the fused features from the dual-branch network and the features from the single-branch network. After dimensionality reduction, the benign and malignant features for were used for comparison. As shown in Figure 11, it will be challenging for classifiers to disentangle the two classes based on the features generated by single-branch networks. In turn, even a very simple classifier could perform well on features extracted with Dual-Net since it exhibits clearer inter-class separation and intra-class clustering.

##### 3.3.3.2 Grad-CAM visualization

In addition to quantitative comparisons, we conducted explainability analysis to understand model behaviors. We visualized their attention patterns using Grad-CAM (48) and overlaid these heatmaps on the original images.

**Figure 12:**
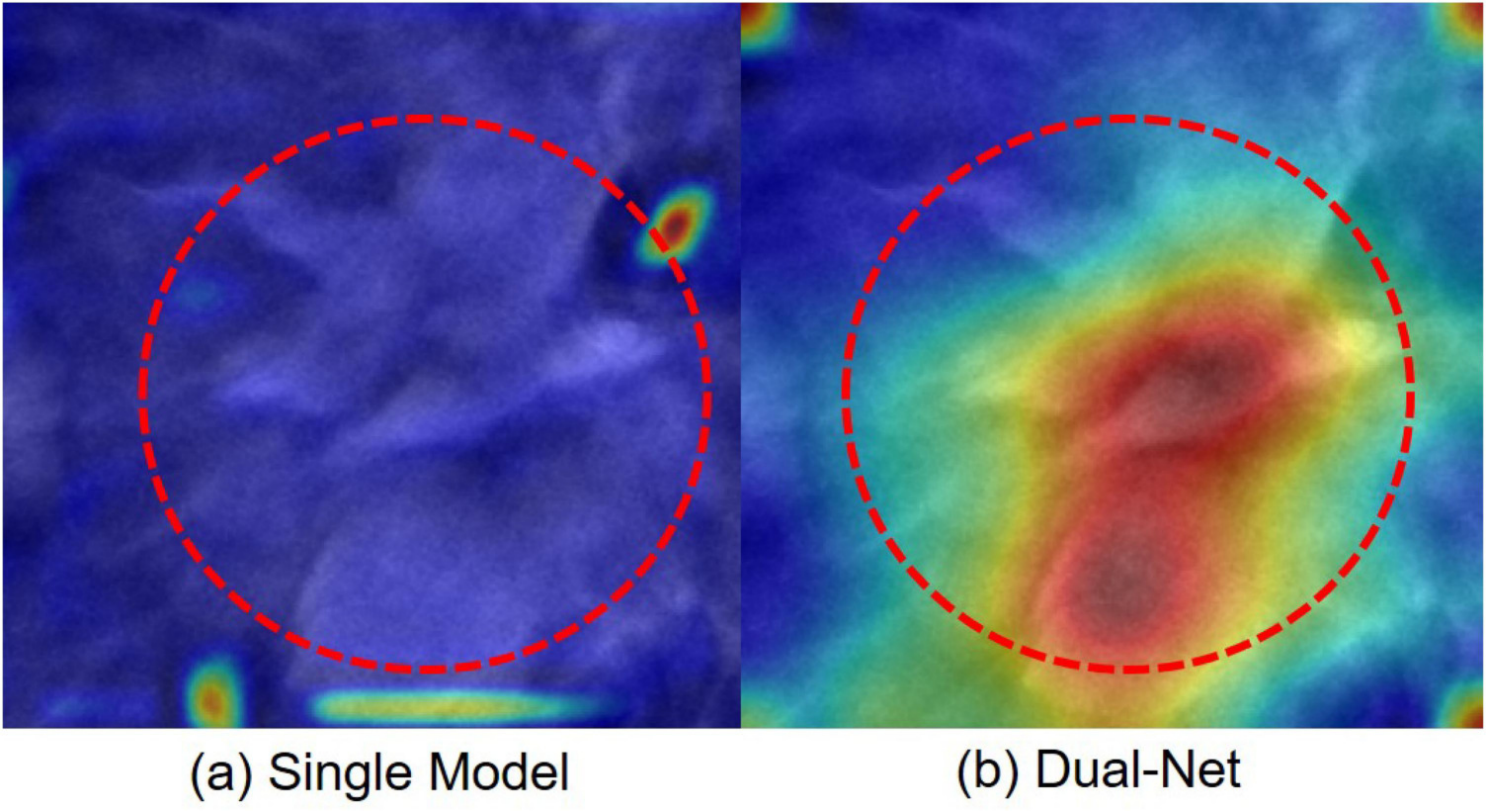
Attention maps of one case misclassified by a single-branch network but correctly classified by our Dual-Net. The red circle indicates the lesion region. **(a)** Single-branch (DenseNet121) Grad-CAM highlights false-positive regions. **(b)** Dual-Net Grad-CAM attention regions.

For Dual-Net, we independently extract feature maps from the final convolutional layer of each branch. Following the computation of gradients on these feature maps, a direct summation strategy to fuse the weighted feature maps from both branches, ultimately projecting the results onto the original image through chromatic mapping. As shown in Figure 12, a representative case illustrates how our model integrates complementary attention patterns from both branches. In this example, we can see that, unlike DenseNet121, Dual-Net focuses on anatomically plausible areas. Thus, the dual-branch architecture attended to more clinically relevant regions.

## 4 Discussion

In this work, we proposed a PCE-based Dual-Net to classify lesions in DBT. The results show the effectiveness of PCE pre-processing in DBT diagnosis. This is in line with several studies that have shown that PCE can improve the medical imaging diagnosis performance (35, 36). We hypothesize that PCE alters the distribution of intensity, which enables the model to make more accurate diagnoses. Psychophysical studies have shown that humans can distinguish between approximately 1–10 million colors, but only around 1,000 gray scales (49). Thus, humans can benefit from using PCE. In clinical practice, pseudo-color can address the shortcomings of grayscale displays and has been widely applied in various clinical digital image analyses (50). For example, PCE can markedly enhance the morphological information of maxillary tumors in oral images and make hidden, millet-sized lesions in thyroid spiral CT images clearly visible. Other applications include multimodal overlays, such as overlaying pseudo-colored PET on CT anatomy to conveniently display functional–structural relationships. While PCE is beneficial for visual interpretation performed by humans, the advantages of using PCE as a pre-processing step for deep learning models are less clear. Indeed, any potential mapping to pseudo-colors would become embedded within the neural network, and, in itself, PCE does not provide additional information that could be useful for the model. Still, our results show that PCE is effective, aligning with previous studies. Even simpler image enhancement methods, such as histogram equalization, yielded improved results. We hypothesize that PCE is effective in cases with limited training data, which is typically the case in medical imaging applications. For sufficiently large datasets, the network can learn richer representations, where pre-processing steps, such as PCE, are expected to have a lesser impact.

### 4.1 Limitations

There are several critical aspects for further investigation. First, the selection of an appropriate backbone network for Dual-Net is crucial for performance. Through comparative experiments, we found that the combination of AlexNet and ResNet achieved the best performance. The result is consistent not only with our experimental observations but also with previous studies, which highlight the importance of low-level features from shallow networks in computer vision tasks (51, 52). In terms of model architecture, we fused two models to capture both low-level and high-level representations, leveraging the complementary strengths of shallow and deep networks. The combinations of CNNs-ViTs and ViT models are also used for comparison. However, methods that included ViTs did not outperform CNN-only models. We attribute it to the limited size of our dataset. Compared to ViT, CNNs have local inductive biases that might help them converge faster in datasets with a limited amount of data (53–55). While the AlexNet–ResNet model performed well on the datasets, its generalization remains constrained by dataset limitations. Future work will focus on expanding the dataset to validate robustness, which may also unlock the potential of more complex architectures, such as ResNet–ViT (54).

Second, at the image level prediction, since each lesion is only annotated with a single slice, we assume each lesion region is spherical in shape. This assumption is common in DBT imaging evaluations (56, 57) or physical phantom simulations (58). However, several studies report that only about 18% of tumors are approximately spherical (53, 60), while many present as discoid or irregular shapes. Thus, our assumption of spherical lesion shape is not fully accurate. However, its impact on the results is expected to be minimal. This is because we chose the smallest lesion in the dataset–10 mm in our case–to estimate the number of slices that are considered for processing in all cases, which corresponds to 10 slices. The probability of having slices without lesion tissue largely decreases with the size of the lesion. Furthermore, in DBT, reconstruction artifacts typically make lesion regions appear larger than their actual size. We manually assessed 10 slices close to the smallest lesions and found lesion tissue in all of them. Figure 7, which visualizes the slices surrounding the smallest lesion, shows that all slices contain lesion tissue. We uniformly set the maximum lesion thickness to 10 slices, determined by the smallest lesion thickness in the dataset. Although this choice does not perfectly reflect the true distribution of lesion sizes, it allowed us to maximize data utilization in a dataset where lesion samples are relatively scarce. In future studies with larger datasets, more flexible sampling strategies that better capture thickness variability will be considered.

Last but not least, we acknowledge some methods from the literature not included for comparison. This limitation stems from two practical considerations: (1) many referenced methods were tested on private datasets, making fair comparisons infeasible; (2) incomplete implementation details in existing works hinder faithful reproduction of their results. At the moment, public DBT data is scarce. In the future, we will validate our method with additional datasets.

## 5 Conclusion

In summary, we proposed a Dual-Net model for DBT lesion classification, integrating a dual-branch network feature leveraging complementary representations from two distinct networks. We systematically evaluated the performance of different deep learning architectures for the classification of benign and malignant lesions in DBT by using the public BCS-DBT dataset. Under identical experimental settings, our approach demonstrated superior classification performance compared to baseline models.

## Data Availability

Publicly available datasets were analyzed in this study. This data can be found here: https://www.cancerimagingarchive.net/collection/breast-cancer-screening-dbt/.
